# Acquired Alopecias in Mexican Adults: A Clinical and Epidemiological Study of 1,888 Patients

**DOI:** 10.7759/cureus.101165

**Published:** 2026-01-09

**Authors:** Joel Alejandro Ramírez-Sánchez, Diana Laura Vazquez-Cantu, Marco Antonio Rodriguez-Castellanos, Elizabeth Guevara-Gutiérrez, Arturo Lopez Yañez-Blanco, Luis Enrique Sánchez-Dueñas

**Affiliations:** 1 Department of Dermatologic Surgery, Instituto Dermatológico de Jalisco "Dr. José Barba Rubio", Guadalajara, MEX; 2 Department of Dermatology, Instituto Dermatológico de Jalisco "Dr. José Barba Rubio", Guadalajara, MEX; 3 Department of Research, Instituto Dermatológico de Jalisco "Dr. José Barba Rubio", Guadalajara, MEX; 4 Department of Pediatric Dermatology, Instituto Dermatológico de Jalisco "Dr. José Barba Rubio", Guadalajara, MEX; 5 Department of Trichology, Instituto Dermatológico de Jalisco "Dr. José Barba Rubio", Guadalajara, MEX

**Keywords:** acquired alopecia, alopecia, alopecia areata (aa), androgenetic alopecia, epidemiology, non-scarring alopecia, scarring alopecia

## Abstract

Introduction: Hair loss has emerged as one of the most common reasons for dermatological consultations; however, reported epidemiological data remain variable.

Objective: This study aims to describe the types and clinical-epidemiological characteristics of acquired alopecia in adult patients.

Materials and methods: A retrospective analysis was conducted on patients diagnosed with acquired alopecia at the Instituto Dermatológico de Jalisco "Dr. José Barba Rubio" between January 1, 2018, and December 31, 2022.

Results: A total of 1,888 cases of acquired alopecia were analyzed, representing the largest case series reported nationally to date. The mean age of the patients was 37.07 years. Female patients accounted for 1,065 cases (56.4%), while male patients represented 823 cases (43.6%). Overall, alopecia areata was the most frequent type, accounting for 871 cases (46.13%), followed by androgenetic alopecia (543 cases, 28.76%) and traction alopecia (127 cases, 6.73%). Non-scarring alopecias comprised 1,661 cases (88.0%) of the studied population, whereas scarring alopecias accounted for 227 cases (12.0%). Among scarring alopecias, cases with neutrophilic or mixed inflammatory infiltrates predominated, accounting for 145 cases (63.98%), with a higher prevalence among male patients. In this study, folliculitis decalvans was identified as a major cause of scarring alopecia in the Mexican population, accounting for 64 cases (3.39%) of all alopecia cases in the cohort.

Conclusion: Regional studies are essential to better understand alopecia patterns and to guide tailored diagnostic and management strategies for affected patients.

## Introduction

Alopecias are a group of disorders that cause hair loss in up to 50% of the population at some point in their lives [1-3]. The primary causes of acquired alopecias are related to genetic predisposition, with onset determined by the interaction of various endogenous and exogenous factors acting as triggers [1,3]. According to the American Hair Research Society, alopecias are classified as scarring alopecias (SAs) and non-scarring alopecias (NSAs) [1,4].

NSA results from an inflammatory process affecting the hair follicles; however, once resolved, the follicular integrity is maintained [1,3]. This group includes androgenetic alopecia (AGA), alopecia areata (AA), telogen effluvium (TE), trichotillomania (TTM), anagen effluvium (AE), and traction alopecia (TA) [1,3].

In SA, hair follicles are replaced by fibrotic scar tissue and are classified based on histopathological characteristics and the predominant inflammatory infiltrate, which may be lymphocytic, neutrophilic, or mixed [4]. Among the lymphocytic group, the main entities include lichen planopilaris (LPP), frontal fibrosing alopecia (FFA), fibrosing alopecia in a patterned distribution (FAPD), discoid lupus erythematosus (DLE), and central centrifugal cicatricial alopecia (CCCA). In the neutrophilic and mixed groups, conditions such as folliculitis decalvans (FD), dissecting cellulitis (DC), and acne keloidalis nuchae (AKN) are included [5].

Acquired alopecias can also occur secondary to severe inflammatory damage caused by infections, leading to irreversible follicular loss. Among the diverse clinical entities are tinea capitis (TC), secondary syphilis (SS), autoimmune diseases such as dermatomyositis (DM), trauma (TRAU), pressure alopecia (PA), and Graham Little-Piccardi-Lassueur syndrome (GLPLS), among others [1,6].

The epidemiology of acquired alopecias has been difficult to establish due to their clinical variability and the influence of ethnicity on hair characteristics. To date, only two epidemiological studies have been conducted in Mexico, including 111 and 144 patients, respectively [7,8]. This study aims to describe the types and clinical-epidemiological characteristics of acquired alopecias in adult patients treated at the Instituto Dermatológico de Jalisco "Dr. José Barba Rubio," with the objective of generating region-specific epidemiological evidence that may support clinical decision-making and contribute to public health understanding of alopecia patterns in the Mexican population.

## Materials and methods

Study design and setting

A retrospective, observational study was conducted between January 1, 2018, and December 31, 2022, including adult patients aged ≥18 years with a clinical and/or histopathological diagnosis of acquired alopecia. Patients were treated at the Instituto Dermatológico de Jalisco "Dr. José Barba Rubio," a tertiary referral dermatology center in Mexico.

Data source and de-identification

Electronic medical records were reviewed using the institutional digital database. All data were fully de-identified prior to analysis, and no personally identifiable information (including names, identification numbers, or contact details) was accessed or recorded. Each patient was assigned a unique study code to ensure confidentiality.

Case identification and diagnostic coding

Patients were identified through ICD-10 diagnostic codes corresponding to acquired alopecia. The search strategy included the following terms: “AE”, “AGA”, ”AKN”, “AA”, “AA incognita”, “alopecia”, “anagen alopecia”, “CCCA”, “DC”, “diffuse AA”, “diffuse alopecia”, “DLE”, “FD”, “FADP”, “female-pattern AGA”, “FFA”, “LPP”, “male-pattern AGA”, “NSA”, “perifolliculitis capitis abscedens et suffodiens”, “SA”, “SS”, “TA”, “TC”, “TE”, “total AA”, “total alopecia”, “TTM”, “universal AA”, and “unspecified hair pathology”. Patients with one or more diagnostic codes were included in the study.

Diagnostic consolidation and classification

To ensure diagnostic consistency and avoid misclassification, related diagnoses were consolidated into diagnostic categories. Terms referring to clinical variants, synonyms, or overlapping entities were grouped together according to established dermatological classification criteria in order to standardize terminology and maintain consistency across the analysis.

Diagnoses were subsequently categorized according to etiology and classified as either SAs (cicatricial) or NSAs. Histopathological evaluation was performed when clinically indicated and used to support or confirm the clinical diagnosis.

Variables and data collection

The following variables were collected: age at diagnosis, sex, type of alopecia, and classification as scarring or non-scarring. In cases of multiple diagnoses, each alopecia subtype was recorded and analyzed.

Statistical analysis

Descriptive statistical analyses were performed using standard statistical methods. Continuous variables were summarized as means and ranges, while categorical variables were expressed as absolute frequencies and percentages, reported in N (%) format throughout the Results section. No inferential statistical analyses were performed due to the descriptive nature of the study.

## Results

A total of 1,888 electronic medical records met the inclusion criteria out of 2,263 records with a diagnosis of acquired alopecia. Of these, 1,662 cases (88.03%) corresponded to NSA and 226 cases (11.97%) to SA. The study population consisted of 824 male patients (43.6%) and 1,064 female patients (56.4%), with a mean age of 37 years (range: 18-88).

Among all cases, AA was the most frequent diagnosis, followed by AGA and TA. The distribution of diagnoses by sex, mean age, and frequency is presented in Table 1.

**Table 1:** Clinical and Epidemiological Characteristics of Acquired Alopecias by Type, Sex, and Mean Age Abbreviations (in alphabetical order): AA: Alopecia Areata, AGA: Androgenetic Alopecia, AGA + TE: Androgenetic Alopecia and Telogen Effluvium, AKN: Acne Keloidalis Nuchae, DC: Dissecting Cellulitis, DLE: Discoid Lupus Erythematosus, DM: Dermatomyositis, FD: Folliculitis Decalvans, FFA: Frontal Fibrosing Alopecia, GLPLS: Graham Little-Piccardi-Lassueur Syndrome, LPP: Lichen Planopilaris, O: Overall, PA: Pressure Alopecia, PFA: Patterned Fibrosing Alopecia, SS: Syphilitic Alopecia, TA: Traction Alopecia, TC: Tinea Capitis, TE: Telogen Effluvium, TRAU: Traumatic Alopecia, TTM: Trichotillomania.

Diagnosis	n (%)	Mean Age (Years)	Age Range (Years)
AA overall	871 (46.13)	35	18–82
AA male	362 (41.56)	32	18–82
AA female	509 (58.44)	36	18–74
AGA overall	543 (28.76)	38	18–85
AGA male	300 (55.25)	33	18–75
AGA female	243 (44.75)	44	18–85
TA overall	127 (6.73)	40	18–70
TA male	3 (2.36)	30	21–41
TA female	124 (97.64)	40	18–70
TE overall	80 (4.24)	45	19–74
TE male	1 (1.25)	32	32–32
TE female	79 (98.75)	46	19–74
FD overall	64 (3.39)	36	18–88
FD male	54 (84.37)	35	18–88
FD female	10 (15.63)	41	19–68
AKN overall	51 (2.70)	31	18–66
AKN male	50 (98.04)	31	18–66
AKN female	1 (1.96)	20	20–20
DC overall	36 (1.91)	30	18–46
DC male	33 (91.67)	29	18–42
DC female	3 (8.33)	42	40–46
LPP overall	32 (1.69)	53	24–81
LPP male	6 (18.75)	50	34–62
LPP female	26 (81.25)	53	24–81
FFA overall	28 (1.48)	52	29–69
FFA male	1 (3.57)	69	69–69
FFA female	27 (96.43)	52	29–68
TTM overall	26 (1.38)	39	18–75
TTM male	5 (19.23)	43	20–65
TTM female	21 (80.77)	38	18–75
TC overall	10 (0.53)	34	18–71
TC male	4 (40.00)	32	20–45
TC female	6 (60.00)	36	18–71
DLE overall	9 (0.48)	60	34–74
DLE male	1 (11.11)	67	67–67
DLE female	8 (88.89)	59	34–74
SS overall	3 (0.16)	37	25–49
SS male	3 (100.00)	37	25–49
SS female	0 (0.00)	37	25–49
Total overall	1,888 (100.00)	37	18–88

**Figure 1:**
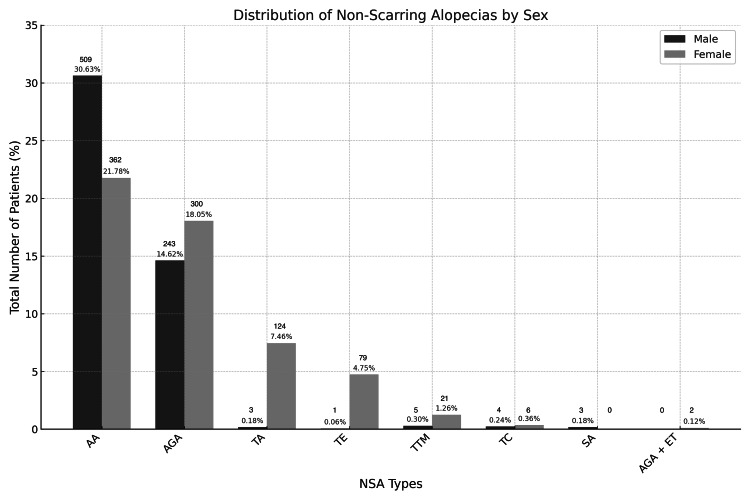
Distribution of Non-Scarring Alopecias by Sex Abbreviations (in alphabetical order): AA: Alopecia Areata, AGA: Androgenetic Alopecia, AGA + TE: Androgenetic Alopecia and Telogen Effluvium, SS: Syphilitic Alopecia, TA: Traction Alopecia, TE: Telogen Effluvium, TC: Tinea Capitis, TTM: Trichotillomania.

Of all NSA cases, AA was the most frequent diagnosis, followed by AGA. The third most common diagnosis differed by sex, with TTM more frequent in males and TA predominating among females. The sex-specific distribution of NSA subtypes is shown in Figure 1.

**Figure 2:**
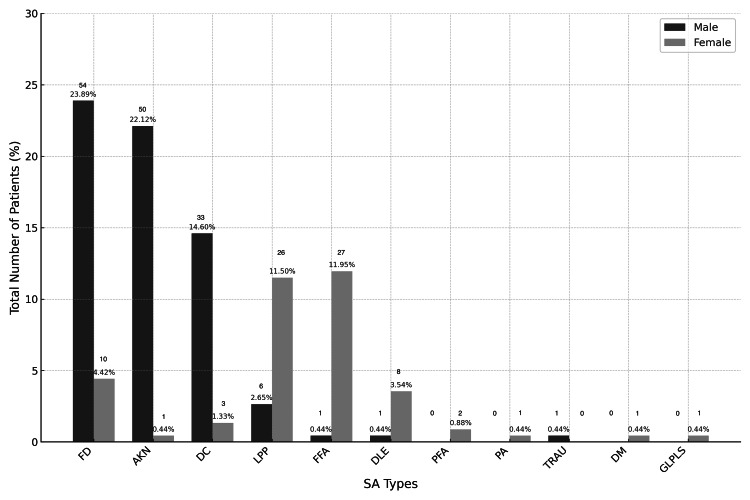
Distribution of Scarring Alopecias by Sex Abbreviations (in alphabetical order): AKN: Acne Keloidalis Nuchae, DC: Dissecting Cellulitis, DLE: Discoid Lupus Erythematosus, DM: Dermatomyositis, FD: Folliculitis Decalvans, FFA: Frontal Fibrosing Alopecia, GLPLS: Graham Little-Piccardi-Lassueur Syndrome, LPP: Lichen Planopilaris, PA: Pressure Alopecia, FAPD: Fibrosing Alopecia in a Pattern Distribution, TRAU: Traumatic Alopecia.

Among the SA cases, the three most common diagnoses were FD (28.32%), AKN (22.57%), and DC (15.93%). Neutrophilic and mixed SA predominated over lymphocytic forms (67.70% vs. 32.30%). Among male patients, the leading causes of SA were FD, AKN, and DC, whereas among female patients, the most frequent diagnoses were FFA, LPP, and FD (Figure 2).

## Discussion

In line with previously published reports, male patients presented for hair disease consultations at a younger age compared with female patients (mean age: 32.76 vs. 40.40 years), despite females accounting for a greater proportion of consultations [8,9]. This sex-related difference in age at presentation has been consistently described in the literature and may reflect an earlier onset of certain alopecias in men, as well as greater healthcare-seeking behavior among women.

NSA accounted for the majority of acquired alopecia cases in our cohort, which is consistent with epidemiological studies reporting NSA frequencies ranging from 75% to 90% of all alopecia cases [7,10]. In previously published series, AGA has been described as the most frequent diagnosis among NSA [7,10]. In contrast, AA was the leading cause of acquired alopecia in our study and showed a female predominance.

AGA represented the second most frequent diagnosis, predominantly affecting males and presenting at a younger age than reported in some international series. Published studies have described AGA frequencies ranging from 24% to 38%, consistently demonstrating male predominance [7,10]. The higher relative frequency of AA compared with AGA in our cohort may therefore reflect differences in referral patterns, ethnic background, or healthcare-seeking behavior.

With regard to SA, 226 cases (11.97%) were identified. Neutrophilic and mixed inflammatory types predominated, accounting for 153 cases (67.70%), and were observed mainly in male patients, whereas lymphocytic SA accounted for 73 cases (32.30%) and showed a female predominance. Although lymphocytic SA is reported as the most frequent subtype in many international series, studies from Latin American populations have described a different distribution, with neutrophilic and mixed SA exceeding lymphocytic forms by nearly 2:1 [11-13]. Our findings are consistent with this regional pattern.

In this Mexican cohort, FD was the most frequent cause of SA, accounting for 64 cases (3.39%) of all alopecia diagnoses and 28.32% of SA cases, with a marked male predominance (54 cases; 84.37%), consistent with previous regional reports [10,11,14]. FD was followed by AKN (51 cases; 2.70%) and DC (36 cases; 1.91%), both conditions classically associated with neutrophilic inflammation and predominantly affecting male patients.

Within lymphocytic SA, published reports have identified FFA and LPP as the most frequently reported diagnoses, with frequencies of 10.8% and 7.6%, respectively [10]. In contrast, LPP was the most frequent lymphocytic SA in our cohort (32 cases; 1.69%), followed by FFA (28 cases; 1.48%). Both conditions primarily affected female patients and presented after the age of 50, in agreement with previously published data [12,15].

Among secondary acquired alopecias, the most frequent diagnoses were TC with 10 cases (0.53%) and SS with three cases (0.16%), followed by TRAU and PA with one case each (0.05%). These entities are recognized but uncommon causes of alopecia in adult populations and have been reported in epidemiological and clinical series [8-10].

Other factors that should be considered when interpreting epidemiological patterns include cultural hairstyling practices, which may contribute to the sex-specific predominance of TA by exerting chronic mechanical tension on the scalp [3]. In addition, the broader temporal context of the study period overlaps with the COVID-19 pandemic. SARS-CoV-2 infection and COVID-19 vaccination have been reported as potential triggers of TE, AA, and other hair disorders in clinical and observational studies [16,17]. Furthermore, GLP-1 receptor agonists have recently been discussed in association with hair changes in emerging literature; however, medication exposure was not systematically documented in our dataset, and the use of these agents was likely limited during much of the study period [18]. For these reasons, their contribution could not be assessed in this cohort, although they represent potentially relevant exposures that warrant evaluation in future studies.

Limitations

This study is limited by its retrospective design, which depends on the accuracy and completeness of electronic medical records. Not all diagnoses were confirmed histopathologically, and some cases relied exclusively on clinical assessment. The study was conducted at a single tertiary referral center, which may introduce referral bias and limit generalizability to the broader population. In addition, disease severity, treatment outcomes, and longitudinal follow-up could not be evaluated due to the study design.

## Conclusions

This study provides a comprehensive clinical and epidemiological analysis of acquired alopecias in the adult population of western Mexico, based on a large retrospective cohort. NSA was the most prevalent group, with AA as the leading diagnosis, followed by AGA and TA. Among SA, FD was the most frequent subtype and predominantly affected male patients, whereas lymphocytic SA, including LPP and FFA, showed a female predominance.

These findings contribute to the understanding of alopecia patterns in the Mexican population and highlight the value of region-specific epidemiological data to support diagnostic decision-making and guide management strategies tailored to local population characteristics.
